# Racial Differences in Response to Antihypertensive Therapy: Does One Size Fits All?

**Published:** 2010

**Authors:** Ajay K Gupta

**Affiliations:** International Centre for Circulatory Health, Imperial College, London

The world health organization has identified high blood pressure (BP) as one of the most important modifiable risk factors to reduce rapidly escalating burden of cardiovascular (CV) disease. Globally, 13.5% of total premature deaths annually (i.e. 7.6 million deaths) are directly attributed to hypertension.^1^ Randomised clinical trial data are consistent in showing that BP reduction substantially reduces cardiovascular morbidity and mortality.^2^ However, despite these facts and widespread availability of effective antihypertensive medications, the vast majority of more than 1 billion hypertensive patients remain with uncontrolled BP.^3^ Even among hypertensive patients who receive treatment, in most countries at least half of them fail to reach currently recommended BP targets.^3^,^4^

Several guidelines in these guidelines are available to help physicians achieve better BP control.^5^–^7^ Most recommendations are derived from evidence generated from clinical trials on Caucasian populations. Therefore unsurprisingly, most guidelines except BHS-NICE guidance,^7^ propose uniform application of these recommendations, regardless of ethnic-origin of the patients. Furthermore, none of these guidelines recommends choice of firstline and/or secondline antihypertensive agents based on phenotypical characteristics (race, age, obesity, and plasma renin activity). Nonetheless, the heterogeneity in BP-lowering response to antihypertensive agents is known for over four decades. In early 1970’s, Laragh classified pathophysiology of essential hypertension into low renin hypertension, and high (or medium) renin hypertension, and suggested that the plasma renin activity levels could be used to predict the BP response to antihypertensive agents.^8^ For example, volume overloaded sub-type of hypertension associated with a low plasma renin activity may benefit from use of diuretics, whereas pre-dominantly vaso-constrictive type of hypertension, associated with a higher plasma renin levels may benefit from the use of a beta-blocker. By extension, the BP- response to antihypertensive therapy may also be predicted by the phenotypic markers of volume overload and sympathetic activity. For example, blacks of African origin have propensity for a higher salt sensitivity, and markedly lower plasma renin levels, compared with whites of Caucasian origin. Whereas, south Asians (and possibly middle-eastern) origin subjects, because of a higher prevalence of central obesity and insulin resistance, are likely to have hypertension mainly driven by a higher sympathetic activity. Equally, age could also serve as a crude marker for plasma renin levels and sympathetic activity, with younger patients responding better to drugs such as angiotensin-converting enzyme (ACE) inhibitors and angiotensinogen receptor blockers(ARB), or a beta-blocker, and older patients responding better to a diuretic or a calcium channel blocker (CCB). Whilst, phenotypical predictors are easier to use in clinical decision making in a routine practice, accurate measurement of plasma renin activity is not a trivial task, as is the measurement of sympathetic activity in a clinical setting. In fact, this may be the reason for limited use of plasma renin activity assays. Furthermore, it is unclear whether a routine use of plasma renin activity assays whilst selecting the first line antihypertensive agents would add significantly to the BP control achieved compared with that achieved using simpler phenotypical correlates, such as race, age and possibly presence of obesity.

Observational studies and clinical trials have shown that commonly used antihypertensive agents exert variable BP- lowering response in ethnic populations. For example, compared to white Caucasians, the black African origin patients exhibit significantly poor BP lowering response to beta-blocker (B drug), ACE inhibitors or ARB’s (A drug), and much better response to CCB (C drug) and diuretics (D drug) when used as monotherapy [9–11]. These findings have been recognised by the BHS-NICE guidelines which recommends choice of first-line agents based on race and age of the patients. Since, <;85% hypertensive patients require more than one drug to achieve BP control, it is equally important to determine whether ethnic (racial) differences also exists in BP response to addition of 2^nd^ line agents. With this regard there have been two important publications recently.^12^,^13^

New analysis^12^ using database of BP lowering arm of the Anglo-Scandinavian Cardiac Outcomes Trial (ASCOT-BPLA)[14], suggests that clinically significant ethnic differences in BP-lowering response exists to both first -and second- line antihypertensive agents. In these analyses, differences in BP response between white, black and south Asian patients with hypertension on a beta-blocker or CCB as monotherapy and a diuretic or ACE inhibitor (perindopril) as second line therapy patients were evaluated among hypertensive patients from the UK arm of ASCOT-BPLA. Serial BP data on 4683 (4348 white, 203 black, 132 south Asian) patients were used for the analysis of monotherapy response (i.e. amlodipine vs. atenolol), and 2794 (2583 white, 129 black, 82 south Asian) patients were included for the analysis of dual therapy response (i.e. perindopril vs. thiazide). On multivariable linear regression, among those who received atenolol monotherapy, black patients were significantly less responsive [Systolic BP (SBP) difference +1.74 (95% CI: -1.1 to 4.6) mm Hg] to therapy compared to white patients (referent). In contrast, on amlodipine monotherapy BP response among the three ethnic groups did not differ significantly. On adding a diuretic to atenolol, BP- lowering was similar among the three ethnic groups. However, on addition of perindopril to amlodipine monotherapy, BP-lowering response differed significantly: compared to whites, black patients had a lesser response (SBP difference: 0.8 (-2.5 to 4.2) mm Hg) and south Asians had a greater blood pressure lowering response (SBP difference -6.2 (-10.2 to -2.2) mm Hg) (interaction test p=0.004). These results are further supported from data from another study, where plasma renin activity significantly predicted the response to both first- and add-on second- line agents, and hence, provides patho-physiological context to Gupta et al. findings.^12^

These new studies have unequivocally shown that there are clinically important differences in BP-lowering response to both first- and second-line antihypertensive agents among hypertensive patients from different ethnic groups. These data illustrates the limitations in interpreting the results of data arising mainly from Caucasian populations to other ethnic groups, such as south-Asians and middle-Eastern origin patients. It also highlights urgent need of conducting clinical trials on patients from other ethnic groups. Pending these results, it is fair to suggest that current data on the use of antihypertensive agents suggest that one size do not fits all, and local (or regional) groups should modify these international guidelines for native consumption. Equally, it is important for the major guideline groups to promote the concept of heterogeneity in response to medications, and provide BP treatment algorithms which take into account patient specific conditions such as race, age, presence of obesity and other co-morbidities, whilst making a choice of first and second line agents for secondary prevention issues.
